# Energy Harvesting from a Thermoelectric Zinc Antimonide Thin Film under Steady and Unsteady Operating Conditions

**DOI:** 10.3390/ma11122365

**Published:** 2018-11-24

**Authors:** Mojtaba Mirhosseini, Alireza Rezania, Bo Iversen, Lasse Rosendahl

**Affiliations:** 1 Department of Energy Technology, Aalborg University, Pontoppidanstraede 111, DK-9220 Aalborg East, Denmark; seh@et.aau.dk (M.M.); lar@et.aau.dk (L.R.); 2 Centre for Materials Crystallography, Department of Chemistry and iNANO, Aarhus University, Langelandsgade 140, DK-8000 Aarhus C, Denmark; bo@chem.au.dk

**Keywords:** thermal cycling, thin film thermoelectric generator (TFTEG), electrical load cycling, transient behavior, zinc antimonide, semiconductor

## Abstract

In practice, there are some considerations to study stability, reliability, and output power optimization of a thermoelectric thin film operating dynamically. In this study stability and performance of a zinc antimonide thin film thermoelectric (TE) specimen is evaluated under transient with thermal and electrical load conditions. Thermoelectric behavior of the specimen and captured energy in each part of a thermal cycle are investigated. Glass is used as the substrate of the thin film, where the heat flow is parallel to the length of the thermoelectric element. In this work, the thermoelectric specimen is fixed between a heat sink exposed to the ambient temperature and a heater block. The specimen is tested under various electrical load cycles during a wide range of thermal cycles. The thermal cycles are provided for five different aimed temperatures at the hot junction, from 160 to 350 °C. The results show that the specimen generates approximately 30% of its total electrical energy during the cooling stage and 70% during the heating stage. The thin film generates maximum power of 8.78, 15.73, 27.81, 42.13, and 60.74 kW per unit volume of the thermoelectric material (kW/m^3^), excluding the substrate, corresponding to hot side temperature of 160, 200, 250, 300, and 350 °C, respectively. Furthermore, the results indicate that the thin film has high reliability after about one thousand thermal and electrical cycles, whereas there is no performance degradation.

## 1. Introduction

Thermoelectric systems have been implemented from many years ago as a credible and reliable technology of energy conversion for various applications such as conversion of the thermal energy into electricity directly as power generation and cooling systems [1,2]. The thermoelectric (TE) material’s efficiency is investigated by using the dimensionless figure of merit as ZT = α^2^ T/(ρ·κ), where α is the Seebeck coefficient, T is the absolute temperature, ρ is the electrical resistivity, and κ is the total thermal conductivity, involving the lattice effect, κ_L_, and the charge carrier effect, κ_e_ [3,4].

Although most engineers and researchers consider bulk materials for high-scale applications, such as waste heat recovery from high temperature industrial furnaces and concentrated solar energy [5,6], thin films’ low power applications are attractively evident. The nanostructure of the thin films causes the inherent phonon scattering that can improve TE properties [7]. ZnSb consists of nontoxic and relatively cheap materials as a novel kind of environmentally and friendly materials. The Zn-Sb binary material is made of ZnSb and β-Zn_4_Sb_3_ which are promising *P*-type thermoelectric materials for low-cost energy production, intended to use at intermediate range of temperatures (between room temperature and 350 °C) [8]. Thin film technique can be an appropriate method for upgrading the properties of thermoelectric materials due to low dimensional quantum confinement [9,10,11,12,13]. The properties improvement of thin film TE materials has introduced a great potential in different applications such as miniaturized sensors, micro power sources, and other low power applications. Moreover, in some applications of micro-scale TE systems, thin films are very efficient [14]. 

In a conventional thin film thermoelectric generator (TFTEG), vertical running of heat flow to the film surface is widely used. In this procedure, the cold side and the hot side are only separated by the film thickness. Nevertheless, the cold side temperature increases promptly by the heat transferred from the hot side, that causes the temperature difference between both sides will greatly diminish in a very short time. According to the small temperature difference, conventional TFTEGs usually have low power output even if the materials figure of merit utilized for thin films is high. Although, the cold area can be cooled to yield the aimed temperature difference, but cooling is not obviously efficient from the viewpoint of energy economics. In a conceptual design, a suggestion for producing more electrical power by implementing TFTEG module is that heat flow propagates parallel to the thin film specimen’s surface. 

Fan et al. [15] represented the characteristics of their designed TFTEGs where, somehow, the heat flow runs in longitudinal direction of the thin film. In their study, the maximum difference of temperature between both sides is considered 85 °C. *P*-type Sb_2_Te_3_ and *N*-type Bi_2_Te_3_ thin films were deposited on glass substrates. They reported that the thin film TEG performance via this structure can be further enhanced by optimizing fabrication methods and TE materials.

In the other study, Fan et al. [16] demonstrated a promising flexible TFTEG using *N*-type Al-doped ZnO and *P*-type Zn-Sb based thin film. The cold side was set to 27 °C, while the hot side was heated by an electrical heater to be maintained at fixed temperatures. Raising the hot side temperature leads to the increase of voltage of the *P-N* junction. Since the temperature reached 230 °C, the voltage had its highest value. However, for the hot side temperature higher than 235 °C, the output voltage immediately decreased. They suggested that the flexible substrate used in the thin film structure should only be applied for operating temperatures under 247 °C. With a further increase of the temperature, the flexible substrate is metamorphosed and the thermal stress across the substrate and thin film TE material is increased, which causes the contact to become loose or the electrode falls off. Mirhosseini et al. [17] experimentally investigated a zinc antimonide thin film thermoelectric uni-leg in a wide range of thermal conditions. The results in steady state condition showed that the Seebeck coefficient increases to a maximum magnitude and then decreases as the hot side temperature increases further. Additionally, they argued that electrical conductivity improvement at contact regions can seriously reduce the waste energy.

Few studies have been carried out to find properties of thin film thermoelectric specimens via parallel heat flow in longitudinal direction by applying fixed hot side temperatures or thermal cycling, since the cold side is set to the ambient temperature.

Fundamental investigations help to find behavior and characteristics of the uni-leg thin film specimen. Based on the obtained results, an efficient TFTEG module by *P-N* couples can be designed and fabricated. Accordingly, in the present research, the zinc antimonide thin film specimen is experimentally tested under different thermal and electrical load conditions. Hence, the specimen is mounted between two blocks—a cold side and hot side—since the cold side block is kept at ambient temperature and the hot side block contacts an electrical heater with the capability of controlling the temperature. The ambient temperature is controlled by a thermostat and is kept in about 30 °C. The heat flow propagates parallel to the thin film surface. Since, operating conditions can practically be unsteady; the analysis is carried out in unsteady state condition as well as steady state. Thermal cycling can be an appropriate way to expose the specimen to different thermal conditions. Changing the hot side temperature by thermal cycling has two stages in each cycle. In the heating stage, the hot side of the sample is heated for 8 min to reach steady state in each aimed temperature. Afterward the heater is turned off in the cooling stage, and the temperature of the hot side starts to decrease for 8 min. Different maximum temperatures (aimed temperatures) on the hot side are provided; 160, 200, 250, 300, and 350 °C, separately in a cyclic manner for each experiment. Two scenarios are planned to test the specimen in presence of electrical load cycles as follow: with stepwise constant electrical load related to peak power output individually for each temperature case, and also with a variable cyclic electrical load.

## 2. Experimental Apparatus and Procedures

### 2.1. Thin Film Fabrication

**Figure 1:**
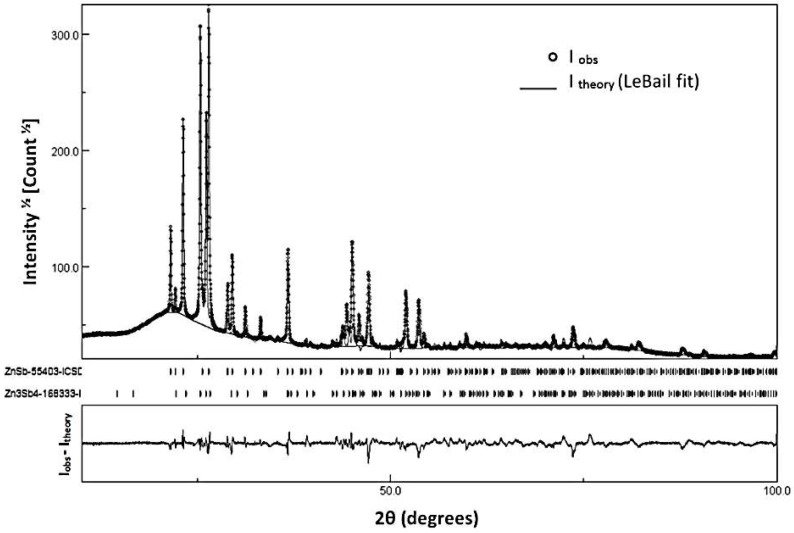
PXRD pattern of the pristine Zn-Sb thin film.

The thin film specimen is fabricated at Aarhus University, Aarhus, Denmark by the magnetron co-sputtering deposition method. The zinc antimonide thin film is directly deposited on a fused silica substrate. The substrate (single-sided polished fused silica wafer) possesses the thickness of 350 μm. Additionally, the thickness of thermoelectric material film is about 600 nm. The substrate is heated to 215 °C during the deposition to result the highest possible crystallization conditions of the thermoelectric film. Argon with a purity of 99.9996% is used as sputter gas at a flow rate of 10 sccm while its pressure in the chamber is set at 0.6 Pa during 60-min deposition time. The power for the Zn_4_Sb_3_ and Zn targets are set to 12 W and 4 W, respectively. Moreover, the chamber base pressure is approximately 3 × 10^−5^ Pa. The as-deposited specimen is heated to 300 °C with a heating rate of 100 °C/h and dwelled for 2 h in air. Phase transitions of the film are directly confirmed by powder X-ray diffraction (PXRD, Aarhus University, Aarhus, Østjylland, Denmark) and some further changes in the electrical properties. The as-deposited and annealed sample is characterized by SEM (Nova 600 NanoLab, FEI, Hillsboro, OR, USA) with EDX, so that PXRD are collected on a 9 kW rotating anode in Rigaku Smartlab, Cu-K_α_ source, parallel beam optics in θ–2θ geometry. In Figure 1, the PXRD pattern is depicted for the specimen together with the theoretical PXRD data set based on LeBail fitting. This specimen’s characteristics are approximately the same as for the Zn-Sb film studied by Sun et al. [18].

### 2.2. Thin Film Test Setup

**Figure 2:**
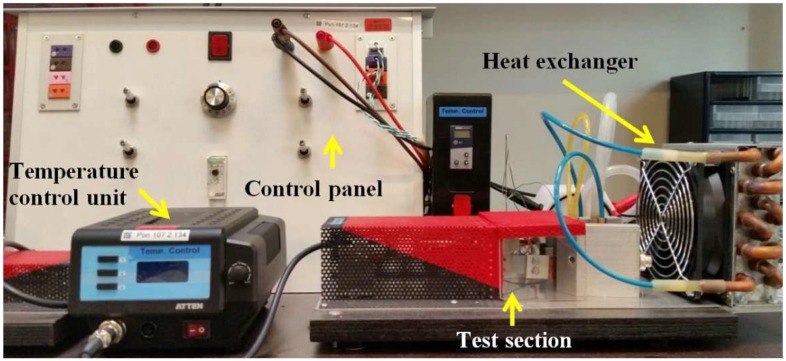
Experimental apparatus.

**Figure 3:**
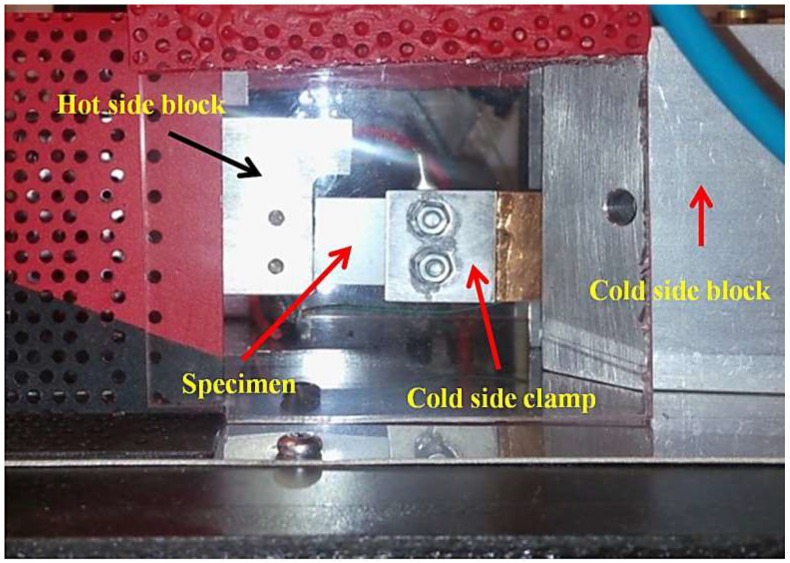
Photograph of the specimen fixed between cold side (**right**) and hot side (**left**) blocks.

The experimental apparatus integrated on the bench (Figure 2) with heating capability of the hot side from 160 to 400 °C. This setup has been designed and developed to test thermoelectric uni-legs and coated thin film specimens. The photograph of the specimen mounted between the hot side and the cold side blocks is shown in Figure 3. The specimen is a rectangular thin film by the length and width equal to 19.8 and 17.2 mm, respectively. The length of hot side and cold side contact area is equal to 3 mm that should be subtracted from the total length to obtaining the effective length. Therefore, the effective length is considered 13.8 mm. The heater switching time is adjustable by a relay placed on the test bench’s control panel to form the thermal cycling. In this research, the heating and cooling time in each cycle for all tests is 8 min, separately. Generally, at the beginning part of the heating stage in each cycle, temperature distribution is transient until temperature of the thin film reaches steady state, before starting the cooling stage. The junctions of the thermoelectric specimen are fixed between two blocks (cold side and hot side). Thermally conductive graphite layers were applied to reduce thermal contact resistance between the specimen and the blocks. More details can be observed in the recent published paper by the same authors [17].

## 3. Results and Discussions

Thermal cycling (without/with electrical load) can indicate how the increase or the decrease of temperature can affect different parameters; and whether these results are reproducible and reliable in the range of permissive standard deviation and with enough precision or not. Herein, to investigate thermoelectric behavior and stability of the sample, voltage and Seebeck coefficient are important parameters which are exhibited for analyzing the open circuit. In addition, the voltage, current, power, and energy generation are main parameters which are studied for closed circuit analysis. All measurements are carried out in 15 s time intervals.

### 3.1. Thermal Cycling without Electrical Load

In this section, thermoelectric characteristics of the specimen are investigated by thermal cycling without applying electrical load resistance. Ten thermal cycles are applied for evaluating repeatability of the results, and to represent the performance consistency of the specimen in operating conditions. The heater switching time is adjustable by a relay placed on the test bench’s control panel to form the thermal cycling. In this research, the heating and cooling time in each cycle for all tests is 8 min, separately. Generally, temperature distribution at the beginning of the heating stage in each cycle is transient until temperature of the thin film reaches equilibrium, before starting the cooling stage. The magnitudes of current and power in this part of experiments are zero, because of the open circuit condition.

**Figure 4:**
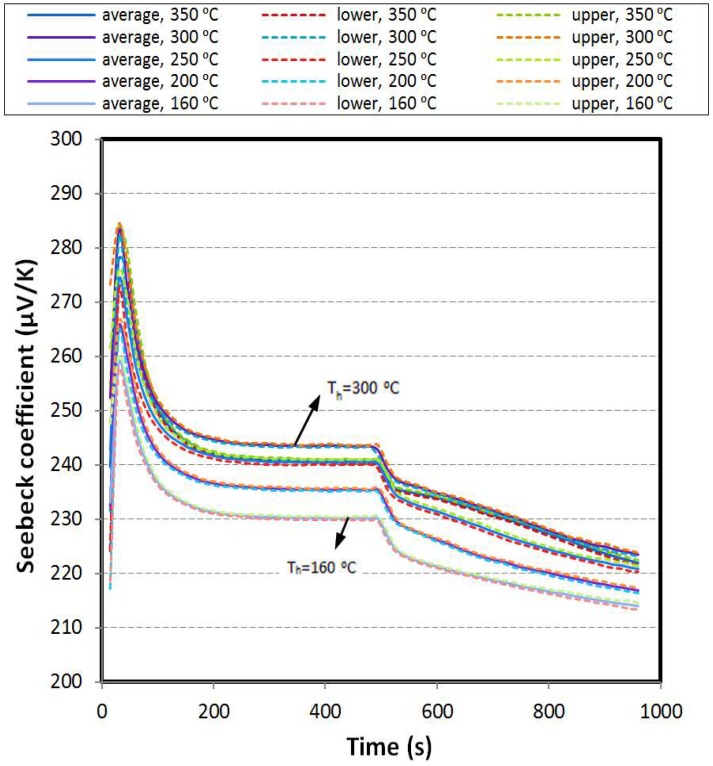
Seebeck coefficient versus time.

Using the voltage and the hot and cold side temperatures to calculate instantaneous value of Seebeck coefficient reveals an interesting trend of the Seebeck coefficient for both transient and steady state conditions. The Seebeck coefficient variation with respect to time is depicted in Figure 4. By tracking the data points in this figure, constant variation shows steady state condition. Except a few data points at the beginning of the heating stage which is for moments of switching on the heater, the temperature difference increasing rate is higher than the rate of increasing the voltage. Therefore, the local Seebeck coefficient is reduced to reach its values at steady state. Moreover, in the cooling stage, the temperature difference degradation rate is less than the voltage decreasing rate and the value of local Seebeck coefficient is again alleviated. During the steady state, these data values have a bit difference to each other, although different thermal boundary conditions are applied for hot side of the specimen. The steady state Seebeck coefficient shows an extremum value. When the hot side temperature increases more than 300 °C, bipolar transport effect occurs. The bipolar effect increases with temperature due to the excitation of electrons from the valence band to the conduction band as the temperature is increased, and create an equal number of holes. These holes and electrons move to the cold side, and transport the heat from the hot side to the cold side of the sample [17]. Although, the net electrical current is zero in this movement due to the equal numbers of opposite charges, the presence of both electrons and holes has a negative effect on the Seebeck coefficient. Bipolar transport effect occurs when temperature of the specimen is higher than an individual temperature corresponding to the type of thermoelectric material, impurities in its structure, manufacturing process, etc. In Figure 4, upper and lower bounds of the data have been exploited by using calculated standard deviation. Thus, these bounds show the average value ± standard deviation. The average values of the standard deviation for the Seebeck coefficient are approximately 0.67, 0.68, 1.0, 0.82, and 0.81 μV/K, respectively, for the thermal cycles with aimed temperature of 160, 200, 250, 300, and 350 °C.

**Figure 5:**
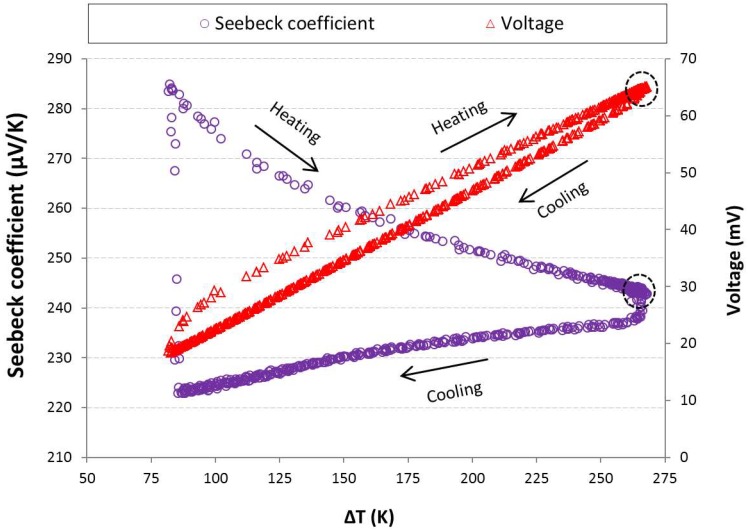
Voltage and Seebeck coefficient versus temperature difference (aimed hot side temperature 300 °C).

In Figure 5, voltage value and Seebeck coefficient versus temperature difference (ΔT = T_hot_ − T_cold_) are shown for hot side boundary condition of 300 °C. There is a similar trend for open circuit results applying other thermal cycles with different temperatures aimed at the hot side. The voltage is increasing since the temperature difference is increased, and vice versa. During the unsteady state, there is not a single voltage data point for each magnitude of temperature difference in heating and cooling stage of a thermal cycle. This is due to several reasons such as hysteresis effect and thermal diffusivity which can change by different parameters. The hysteresis magnitude depends on various parameters such as particle size, thickness, impurities of the specimen, and rate of heating or cooling. The voltage data points in transient condition are disarticulated, while the data points corresponding to the steady state condition are obviously accumulated. The voltage values in steady state have been demonstrated in the circle in Figure 5.

The steady state Seebeck coefficient (inside the circle) for thermal cycle with the aimed hot side temperature of 300 °C shows the highest magnitude than the results of other thermal cycles. Due to this reason, the data corresponding to this cycle is only represented. The bipolar transport effect reduces the steady state Seebeck coefficient when the hot side temperature is higher than 300 °C (Figure 4). This effect depends to type of the material, its manufacturing process and impurities in its structure, etc. Bipolar phenomenon affects the whole body of the specimen and its total characteristics, such as total Seebeck coefficient and thermal conductivity. As seen in this figure, the value of Seebeck coefficient (α) at steady state is approximately 244 μV/K.

### 3.2. Thermal Cycling with Stepwise Constant Electrical Load

In practice, there are some restrictions that must be considered for stability, reliability and output power optimization purposes when a thin film TE operates dynamically. The aim of this study is to evaluate the performance and estimate the energy that would be captured in heating and cooling stage of a cycle. Ten thermal cycles with stepwise constant electrical load are applied for evaluating the reproducibility of the results and to represent the performance consistency of the specimen under operating conditions. In fact, in this subsection, using constant load in heating or cooling stages are two examples of applying electrical load cycling. A wide range of electrical load resistance (from 10 to 300 Ω) was initially utilized to explore optimal electrical load corresponding to the peak power output by the thin film. Table 1 shows that the obtained load resistance is temperature dependent. In each case, the individual load corresponding to its peak power point (PPP) is applied [17]. In all figures of this subsection the upper and lower bounds of the data have been exploited by using calculated standard deviation. Thus, these bounds show the average values ± standard deviations.

#### 3.2.1. Constant Load over Heating Stage

**Figure 6:**
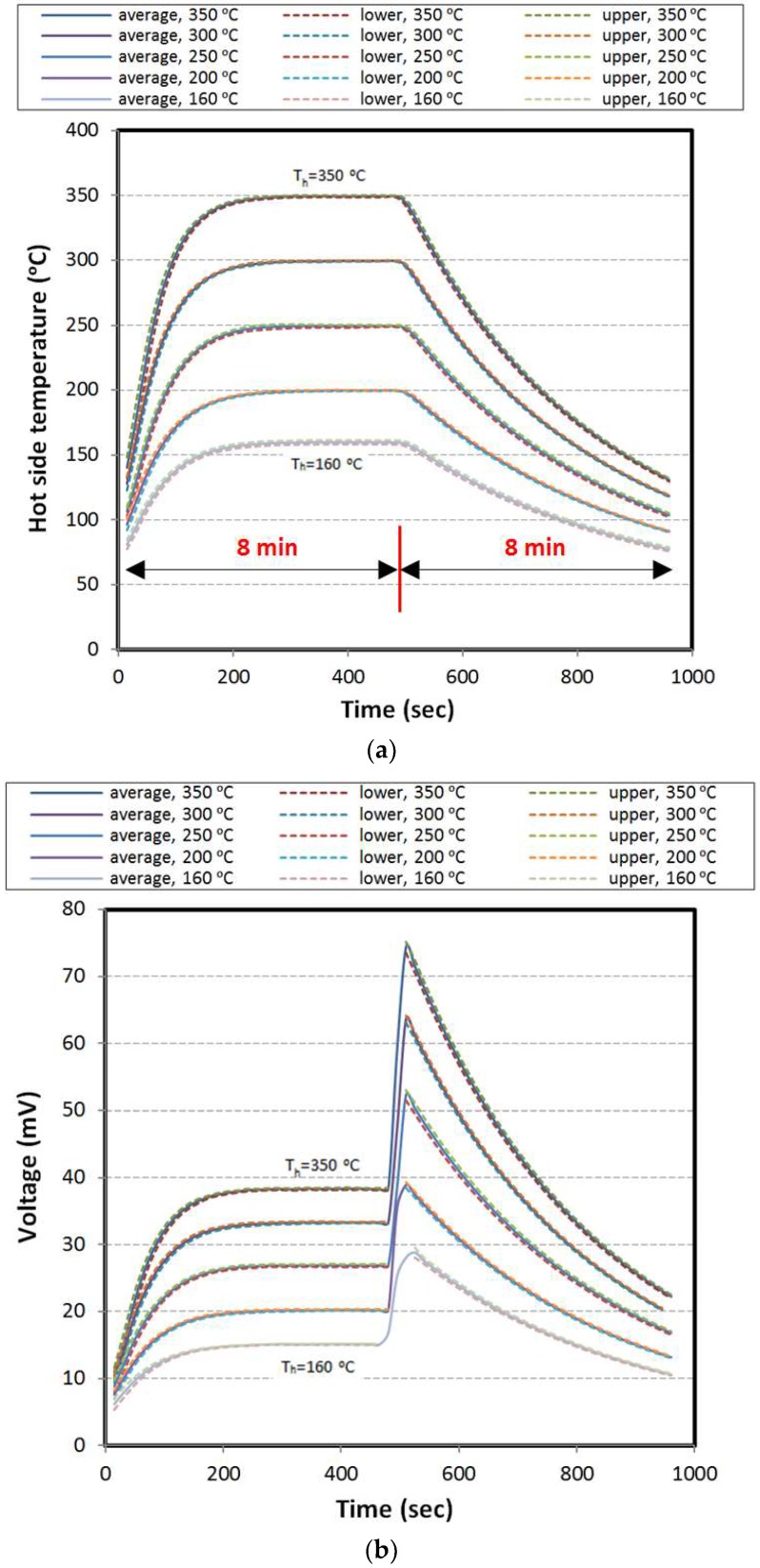

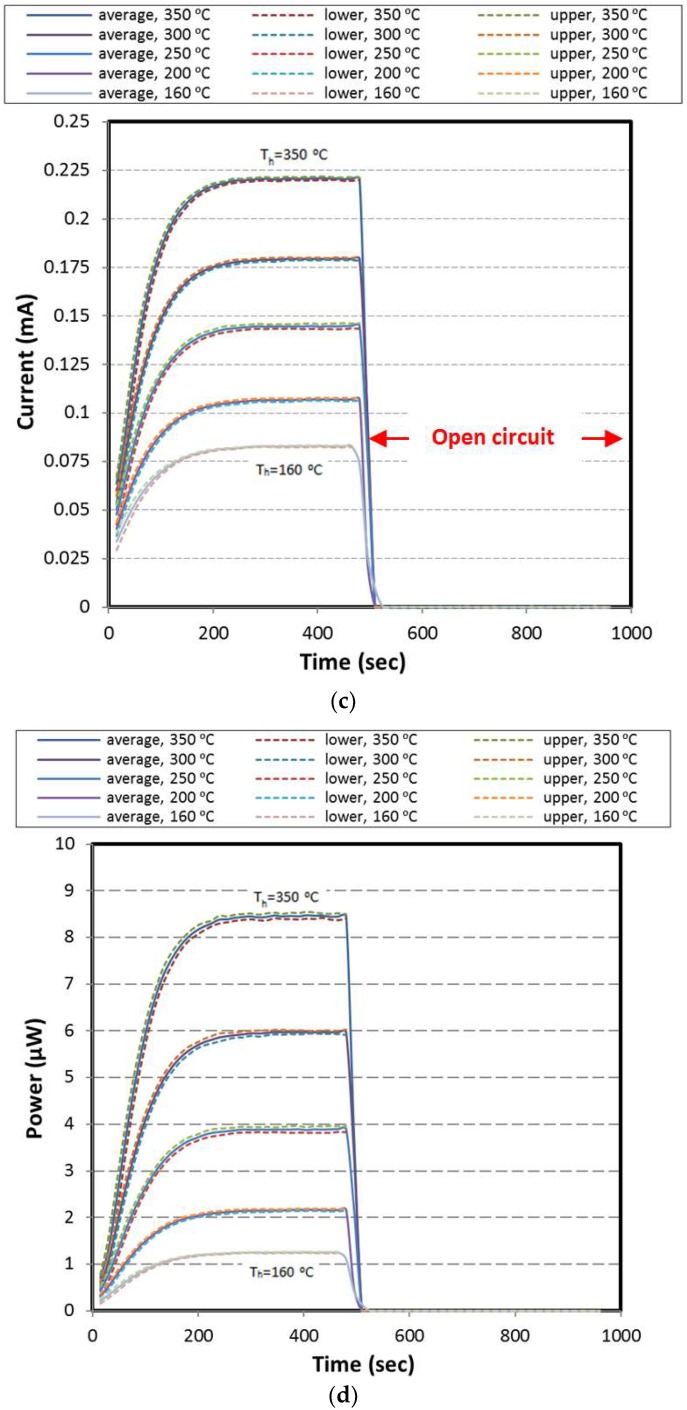
Results for constant load over heating; (**a**) hot side temperature, (**b**) voltage, (**c**) current, and (**d**) power.

In this part, the constant load corresponding to maximum output power, proportional to each hot side condition is applied in the heating stage. The hot side temperature of the specimen with respect to time is shown in Figure 6a. As mentioned, the temperature of the cold side block is kept at the ambient temperature equal to 30 °C.

Obviously, when the hot side temperature of the specimen is increasing, voltage, current, and power are increasing with the same trend until approaching the steady state condition. After eliminating the load, the value of voltage reaches its maximum in open circuit and the current and power reach zero. It should be noted that the rate of voltage reduction in the cooling stage shows a similar trend to the hot side temperature. In Figure 6b–d the results of voltage, current, and power are shown.

#### 3.2.2. Constant Load over Cooling Stage

**Figure 7:**
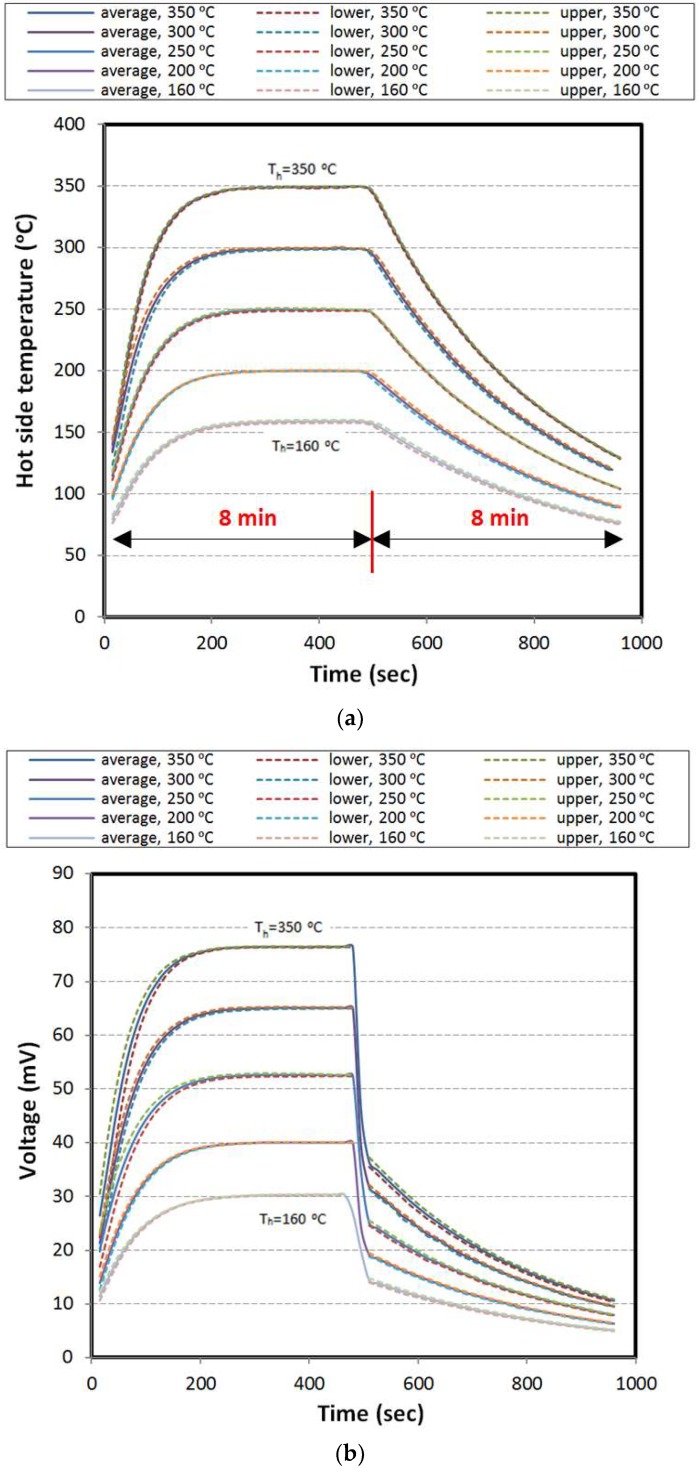

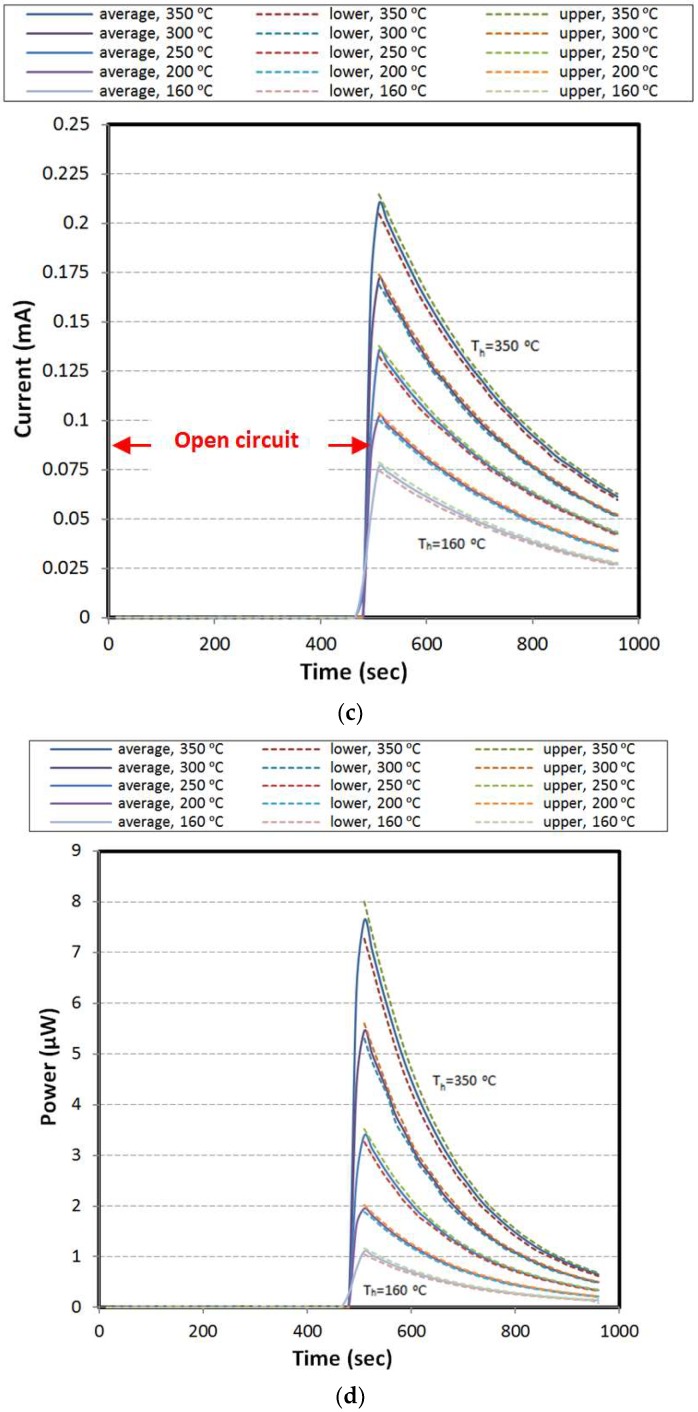
Results for constant load over cooling; (**a**) hot side temperature, (**b**) voltage, (**c**) current, and (**d**) power.

Herein, the electrical load is not applied during the heating stage. The open circuit voltage increases by increasing the hot side temperature until it reaches the steady state. Applying the electrical load exactly at starting points of the cooling stages results dropping in voltage and rising in the current and power. As shown in Figure 7, voltage, current, and power are alleviated in the cooling stage based on the trend of temperature curve. Figure 6b and Figure 7b show that the voltages corresponding to the peak power points are half of the open circuit voltages.

#### 3.2.3. Energy Generation Analysis by Using Constant Load

Calculation and comparison of energy production of the thermoelectric element along the experiments are based on power curves versus time. The trapezoidal rule is used to approximate the area under the power curve versus time. This area identifies the energy generation. By applying the trapezoidal rule to each interval (15 s between two tandem measurements) and summing the results, the produced energy can be estimated with relatively high accuracy. The energy generation is calculated for all thermal cycles; however the average value of ten cycles is shown in Table 2. In this table, energy generation in the case of using load in the heating stage is higher than using the same load in the cooling stage. Almost 70% of the total produced energy belongs to the heating stage, and about 30% of it is for capturing energy in the cooling stage. However, these percentages may change if the period of the cycles or the time involving heating and/or cooling stage change. In contrast to theory, addition of the energies which are separately produced by applying load in heating and cooling stages has a small difference with the energy produced by full loaded cycles in each experiment. It is due to unavoidable errors from the measurement devices and changing the environmental temperature by a deviation of 1–2 °C between different tests. As demonstrated in Table 2, the difference percentage between two styles of calculation is less than 10% (‘*’ is considered as the reference value for calculating the difference percentage). More comparisons can be done according to the contents of this table. The parameter σ shows the standard deviation.

### 3.3. Thermal Cycling with Load Cycling

Thermal cycling is applied similar to the last section for different aimed hot side temperatures. In this section, the applied electrical load changes from the first cycle to the mid cycle step by step from 50 to 300 Ω, and vice versa to the last cycle. The load variation step is 50 Ω. Therefore, in this evaluation, each test possesses eleven thermal cycles.

#### 3.3.1. Different Loads over Heating Stage

**Figure 8:**
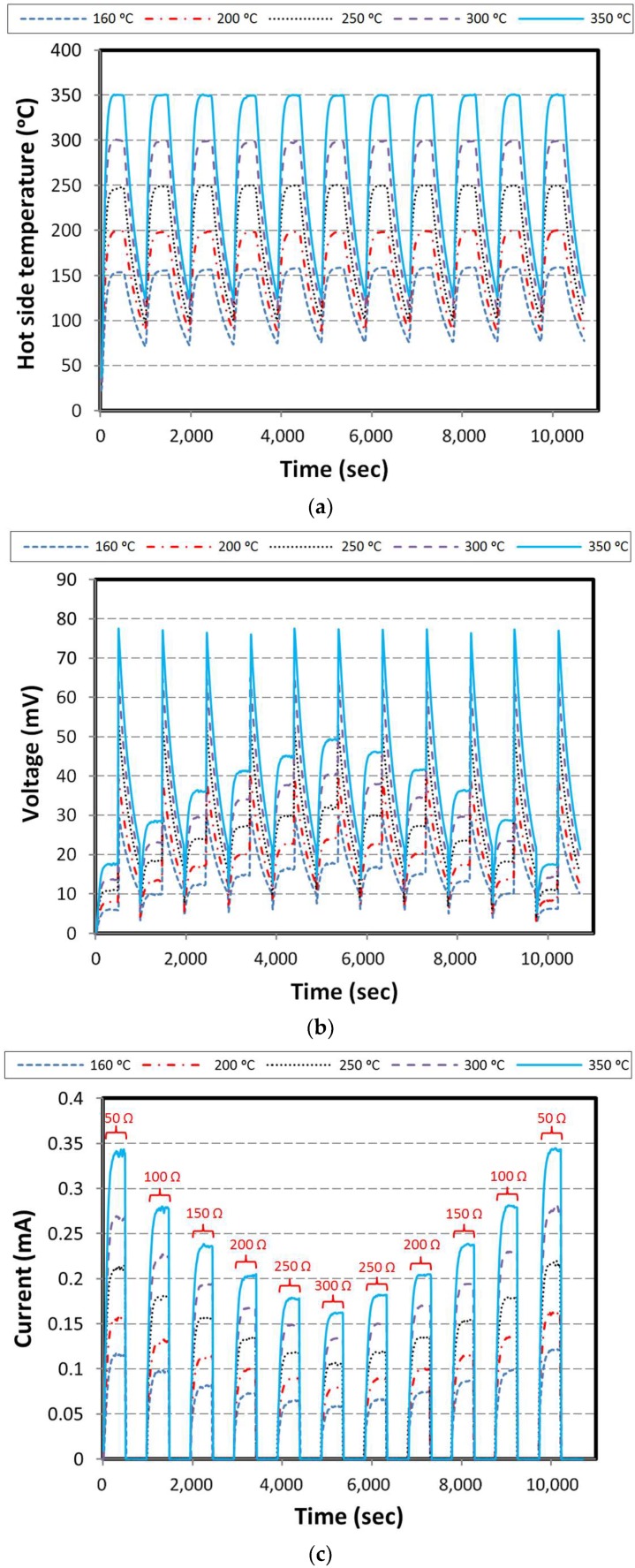

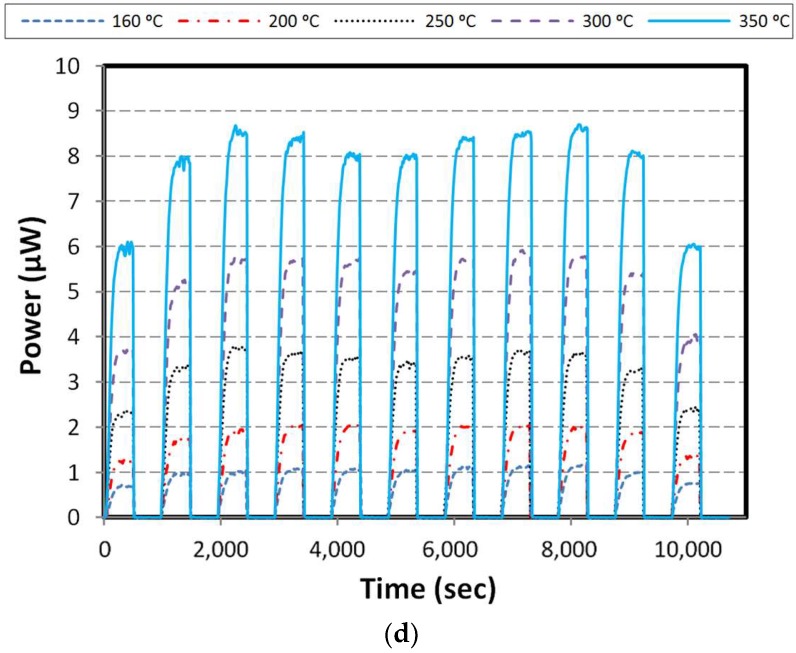
Results for load cycling over heating; (**a**) hot side temperature, (**b**) voltage, (**c**) current, and (**d**) power.

By using load just in the heating stage, the voltage, current, and power are increased with the same behavior of raising the hot side temperature. At beginning point of cooling stage, the load is eliminated that yields the fast increase of the voltage and the decrease of the current and power to zero (open circuit condition). The voltage reduction rate is exactly related to the hot side temperature of its steady state condition. Additionally, at the end points of the cooling stage, the temperatures in different cases have the same transposition which they had at the beginning of the cooling stage. The difference between these points to each other is less than the differences at those first points of the cooling stage. As expected, by applying higher loads in the next cycles rather than the previous ones, the voltage and current increase and decrease, respectively, whereas the power value increases by the load from 50 to its optimal point, about 150 or 200 Ω, and then decreases (Figure 8). The obtained maximum value is closed to the real peak power output at each aimed temperature. The value of peak power point’s load corresponding to each aimed hot side temperature has been mentioned in Table 1. Furthermore, for loads from 300 to 50 Ω in the rest of the thermal cycles, the same results can be obtained as expected. In cycles with the highest aimed hot side temperature (350 °C), the maximum output power happens at load 150 Ω (closer to 170.25 Ω), but in cycles with lowest aimed hot side temperature (160 °C), it happens at 200 Ω (closer to 188.01 Ω). The results confirm the optimal load resistance leading to the maximum power generation in Table 1.

#### 3.3.2. Different Loads over Cooling Stage

**Figure 9:**
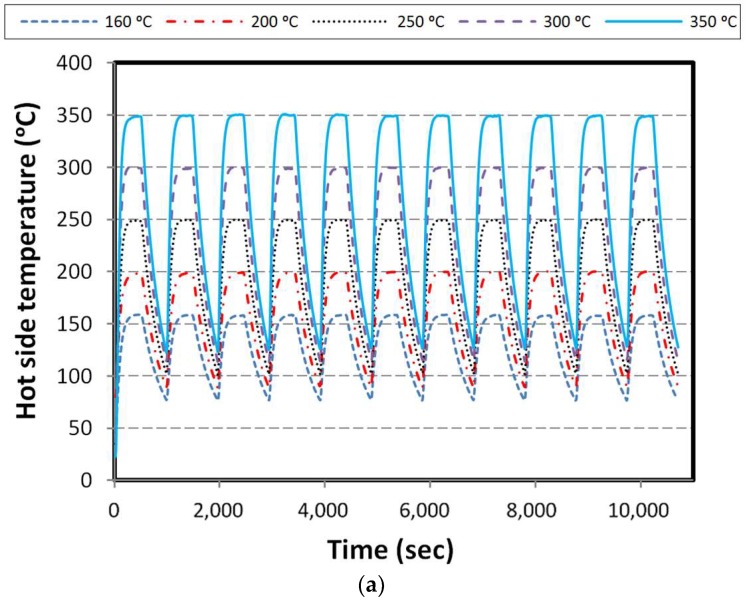

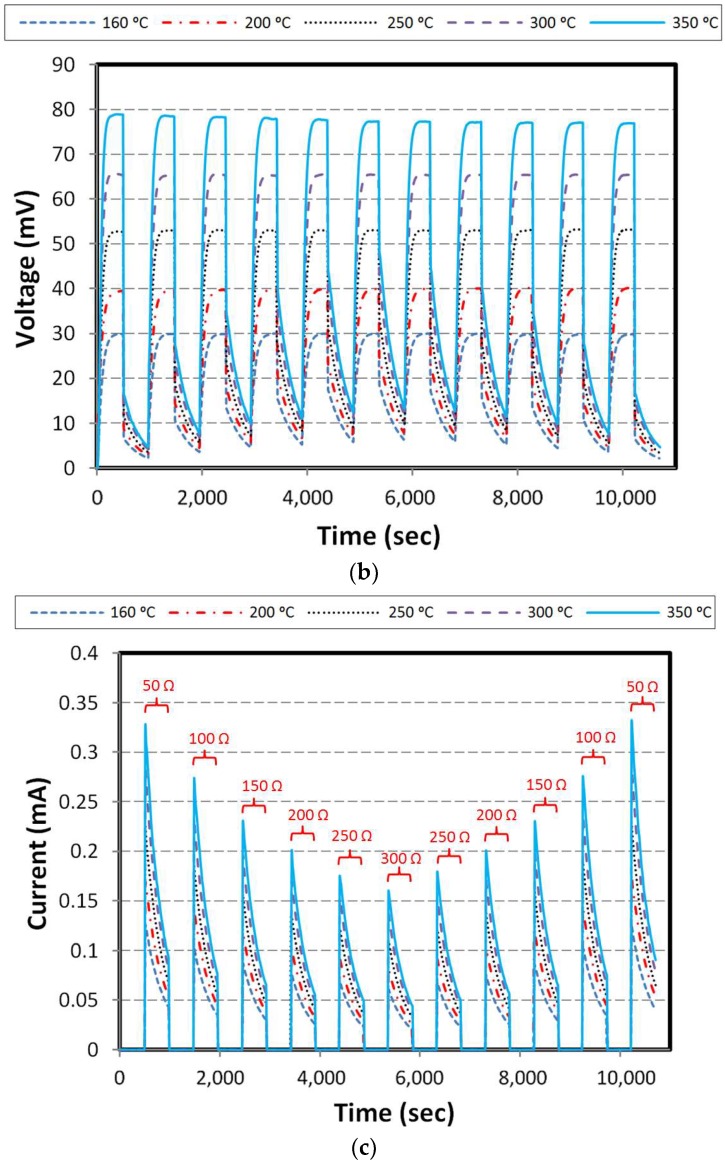

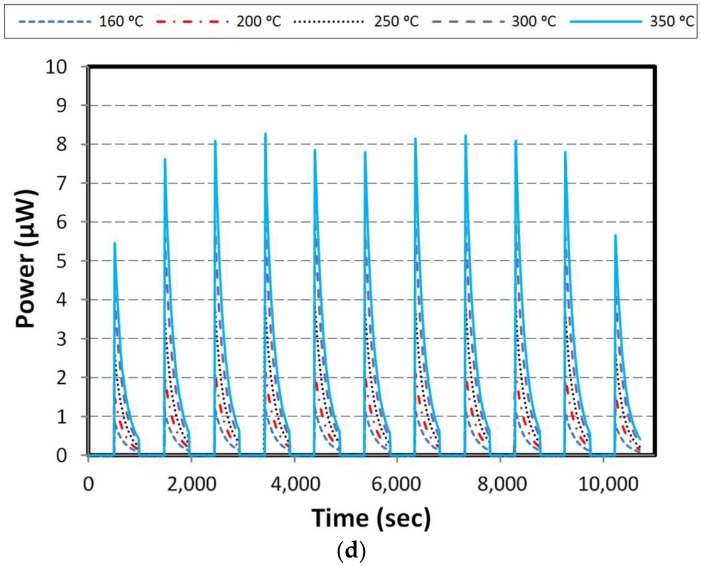
Results for load cycling over cooling; (**a**) hot side temperature, (**b**) voltage, (**c**) current, and (**d**) power.

In this part, the electrical loads are applied in the cooling stage of the cycles step by step from 50 to 300 Ω and vice versa. At the start point of the cooling stage, the applied load causes the voltage decreases while the current and power increases forcefully. As the hot side temperature decreases in the cooling stage, the voltage, current and power reduce, but the rate of this reduction between different cases is not the same with regard to their steady operating conditions. In order to doing more assessment in the cooling stage, for instance it is shown that increasing the load causes the maximum and the minimum values of the voltage increases; in spite of that the maximum and minimum values of the current decreases. Similar to the results obtained by using loads just in heating, herein, the specimen produces power close to its peak power output at load 150–200 Ω (Figure 9).

#### 3.3.3. Different Loads over Cycles

**Figure 10:**
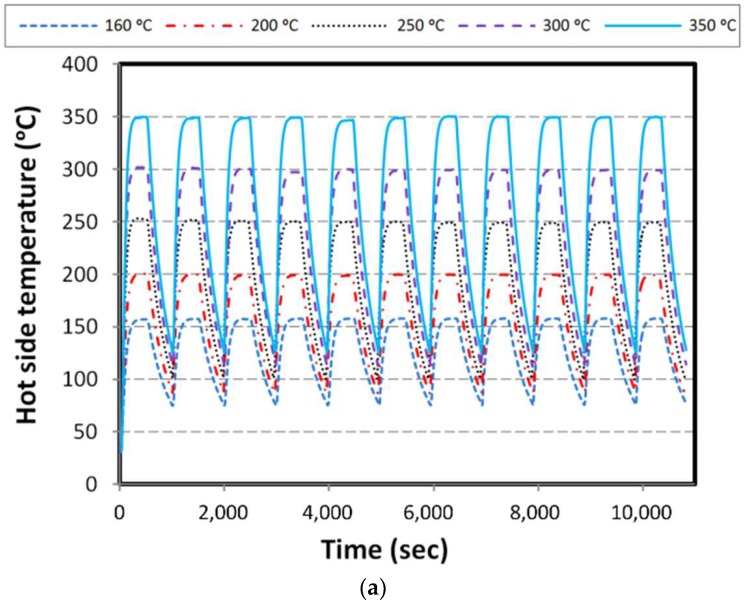

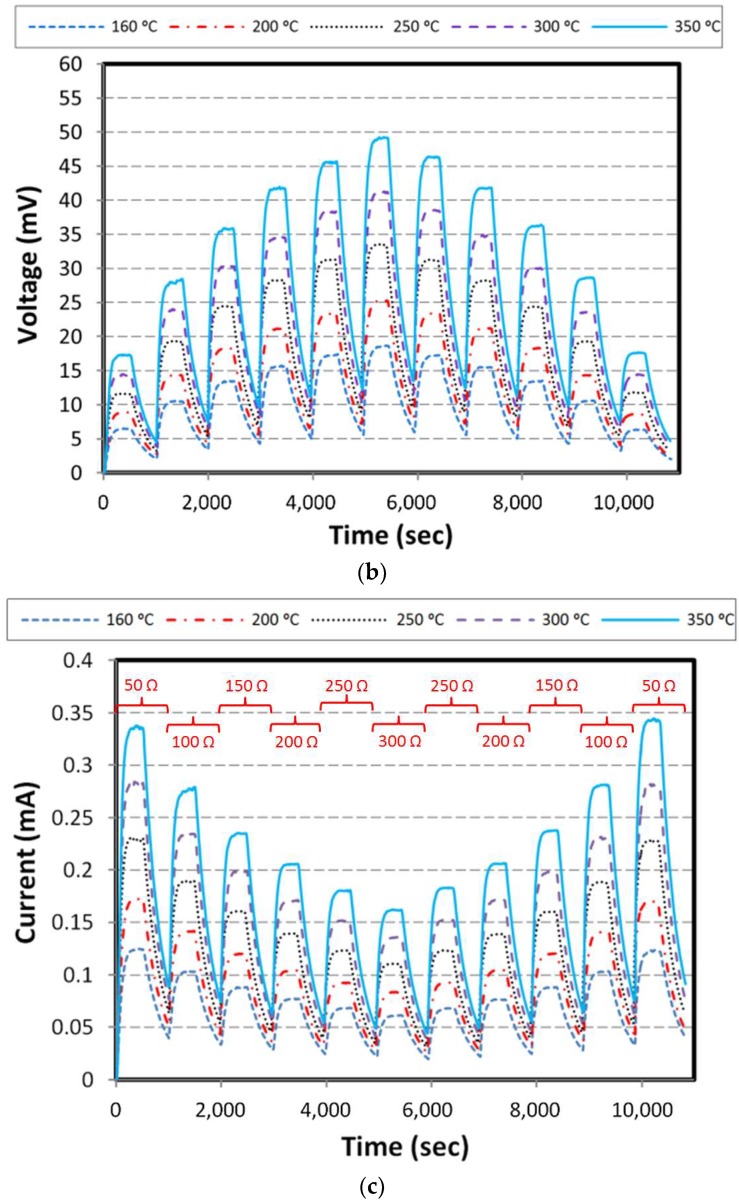

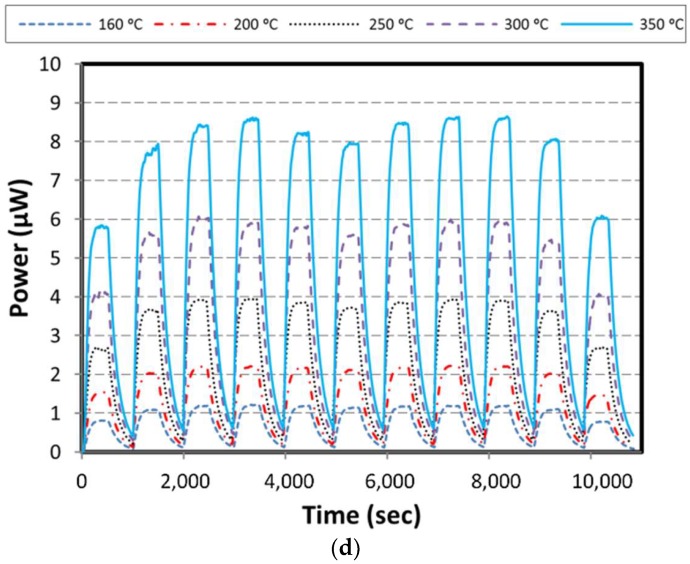
Results for load cycling over cycles; (**a**) hot side temperature, (**b**) voltage, (**c**) current, and (**d**) power.

In this series of experiments, the load is applied from the start point of the thermal cycles, and continues to the end of that experiment. Moreover, theses loads change from 50 to 300 Ω, and vice versa. As shown in Figure 10, variation of the voltage by applying lower load in aimed hot side temperatures is much less than its variation by applying higher load.

In other word, by increasing the electrical load, the difference between the voltage curves is increased, whereas the difference between the current curves decreases. Effect of changing the loads particularly in higher aimed hot side temperatures can be clearly recognized. For instance, in the hot side temperature 160 °C, excepting the first and the last cycle, power generation is more-less constant in response to load variation in comparison with higher aimed temperatures.

The power per unit volume of the thermoelectric material is introduced as power density that can be useful to assess the thin film specimen performance. In Table 3, this parameter is represented for various fixed temperatures on the hot side under steady state conditions.

#### 3.3.4. Energy Generation Analysis by Using Load Cycling

Energy generation in the case of using load cycle in the heating stage is higher than using the same load cycle in the cooling stage. Almost 68% of the total produced energy is related to applying load in the heating stage, and about 32% of it is for capturing power in the cooling stage. Addition of energies which are separately generated in heating and cooling stages has small difference comparing the energy generated by full loaded thermal cycles in the test related to each experiment because of unavoidable errors from the measurement devices and negligible change in the environmental temperature. The maximum percentage of difference between these two ways of calculating the produced energy in full loaded whole cycles is about 6.5–7%, which happens for the cases 160 and 200 °C (Table 4). Herein, the magnitudes of the column, *, are considered as the reference values for difference percentage calculation.

**Figure 11:**
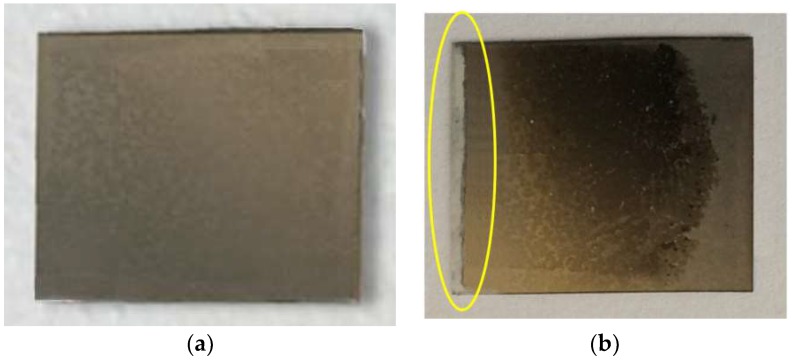
(**a**) The specimen before the tests, and (**b**) the specimen after the tests.

In Figure 11, a picture of the studied specimen is shown. The results indicated that, the thin film has high reliability after about one thousand thermal and electrical cycles, whereas there is no performance degradation during the tests. However, the visual properties of the pristine specimen have changed after the tests, particularly near the hot side edge. It seems the thermoelectric material on the glass substrate has been vaporized in this region due to numerous long-term experiments.

## 4. Conclusions

Behavior of Zn-Sb thin film specimen in transient and steady state conditions is evaluated, so that the heat transfer is in-plane across the thermoelectric element. Effects of applying thermal cycling with various aimed temperatures at hot side of the specimen were investigated in two scenarios; with the stepwise constant electrical load corresponding to the peak power output in each aimed temperature, and with variable cyclic electrical load. The energy generation by applying electrical load in heating stages of the thermal cycles is approximately 70% of the total energy generated by using the same load over entire domain of thermal cycles, while it is approximately 30%, if the cooling stages are just utilized for power generation. The results demonstrated that the data are reproducible and reliable with small magnitudes of standard deviation and well enough precision. By increasing the maximum hot side temperature, the magnitude of standard deviation increases. In the first scenario with one thermal cycle, the whole volumetric energy (energy density) generated by the thermoelectric element is approximately 4.77, 8.52, 15.32, 24.1, and 33.46 mJ/mm^3^, while in the second scenario with 11 thermal cycles, the average values are 4.63, 8.49, 15.03, 22.63, and 32.57 mJ/mm^3^ corresponding to 160, 200, 250, 300, and 350 °C hot side temperature, respectively. The energy density is calculated based on volume of the thermoelectric layer excluding the substrate. One significant message of this study could be that the thin film element by longitudinal direction for heat transfer does not need to have an efficient heat sink. In addition, the thin film specimen can operate without failure in relatively different ranges of temperature and electrical load with long operating period.

## Figures and Tables

**Table 1:** Electrical load resistance corresponding to matched power output by considering contact resistance.

Temperature of Hot Side (°C)	350	300	250	200	160
Electrical load resistance (at PPP) (Ω)	170.25	181.85	182.63	186.51	188.01

**Table 2:** Energy generation in heating stage, cooling stage, and whole cycle (average value in one cycle).

Hot side Temperature (°C)	Heating (J)	Cooling (J)	*Whole Cycle (J)	**(Cooling + Heating) (J)	% Difference (*,**)	Ratio (Heating/**)	Ratio (Cooling/**)
160 (±σ)	5.1821 × 10^−4^ (±1.16 × 10^−5^)	2.29074 × 10^−4^ (±6.1 × 10^−6^)	6.7943 × 10^−4^ (±7.81 × 10^−6^)	7.4728 × 10^−4^	9.99	0.69	0.31
200 (±σ)	8.8666 × 10^−4^ (±1.27 × 10^−5^)	3.87209 × 10^−4^ (±7.59 × 10^−6^)	1.213583 × 10^−3^ (±1.00 × 10^−5^)	1.27387 × 10^−3^	4.97	0.7	0.3
250 (±σ)	1.607357 × 10^−3^ (±3.16 × 10^−5^)	6.54167 × 10^−4^ (±1.25 × 10^−5^)	2.181839 × 10^−3^ (±1.81 × 10^−5^)	2.26152 × 10^−3^	3.65	0.71	0.29
300 (±σ)	2.45647 × 10^−3^ (±3.62 × 10^−5^)	1.00352 × 10^−3^ (±2.05 × 10^−5^)	3.431693 × 10^−3^ (±4.57 × 10^−5^)	3.45999 × 10^−3^	0.82	0.71	0.29
350 (±σ)	3.48041 × 10^−3^ (±4.18 × 10^−4^)	1.396101 × 10^−3^ (±4.46 × 10^−5^)	4.765051 × 10^−3^ (±4.21 × 10^−5^)	4.87651 × 10^−3^	2.34	0.71	0.29

* ** These signs are applied to imply ‘Whole Cycle’ and ‘Cooling and Heating’ in order to avoid repetition.

**Table 3:** Volumetric power (power density) of the zinc antimonide thin film thermoelectric element excluding the substrate, (W/m^3^)

Hot Side Temperature (°C)	Electrical Load Resistance (Ω)					
	50	100	150	200	250	300
160	5898.2	7583.4	8074.9	8285.6	8145.2	7934.5
200	10,181.4	14,043.3	15,237	15,588.1	15,237	14,815.7
250	18,747.8	25,278	27,454.7	27,454.7	27,033.5	25,839.7
300	28,086.7	38,198	41,568.4	41,498.1	41,217.3	39,110.8
350	42,130.1	56,103.2	60,597.1	60,175.8	59,333.2	55,752.2

**Table 4:** Energy generation by load cycling in heating stage, cooling stage, and whole cycles (11 thermal cycles).

Hot Side Temperature (°C)	Heating (J)	Cooling (J)	*Whole cycle (J)	**Cooling + heating (J)	% difference (*,**)	Ratio (Heating/**)	Ratio (Cooling/**)
160	4.446 × 10^−3^	2.3348 × 10^−3^	7.25735 × 10^−3^	6.7808 × 10^−3^	6.57	0.66	0.34
200	8.23511 × 10^−3^	4.1316 × 10^−3^	1.33041 × 10^−2^	1.236671 × 10^−2^	7.05	0.67	0.33
250	1.51476 × 10^−2^	6.91036 × 10^−3^	2.35492 × 10^−2^	2.205796 × 10^−2^	6.33	0.69	0.31
300	2.4006 × 10^−2^	1.06 × 10^−2^	3.545942 × 10^−2^	3.4606 × 10^−2^	2.41	0.69	0.31
350	3.577066 × 10^−2^	1.419233 × 10^−2^	5.1031 × 10^−2^	4.996299 × 10^−2^	2.09	0.72	0.28

* ** These signs are applied to imply ‘Whole Cycle’ and ‘Cooling and Heating’ in order to avoid repetition.
